# Orally Administered Koumine Persists Longer in the Plasma of Aged Rats Than That of Adult Rats as Assessed by Ultra-Performance Liquid Chromatography-Tandem Mass Spectrometry

**DOI:** 10.3389/fphar.2020.01113

**Published:** 2020-07-21

**Authors:** Li-Xiang Ye, Ying Xu, Shui-Hua Zhang, Da-Xuan Cao, Ling-Fan Chen, Yan-Ping Su, Hui-Hui Huang, Chang-Xi Yu

**Affiliations:** ^1^ Fujian Center for Safety Evaluation of New Drug, Fujian Medical University, Fuzhou, China; ^2^ Department of Pharmacology, College of Pharmacy, Fujian Medical University, Fuzhou, China; ^3^ Fujian Key Laboratory of Natural Medicine Pharmacology, College of Pharmacy, Fujian Medical University, Fuzhou, China; ^4^ Fujian Key Laboratory of Drug Target Discovery and Structural and Functional Research, College of Pharmacy, Fujian Medical University, Fuzhou, China

**Keywords:** Koumine, ultra-performance liquid chromatography-tandem mass spectrometry, aging, dose proportionality, pharmacokinetics

## Abstract

Aging leads to changes in nearly all pharmacokinetic phases. Koumine (KM), an alkaloid derived from *Gelsemium elegans* Benth., is effective against age-associated chronic diseases, but its dose proportionality following oral administration in aged individuals remains unknown. Herein, we established and validated a simple method that requires low sample volumes to determine KM concentration in rats using ultra-performance liquid chromatography-tandem mass spectrometry. The maximum plasma concentration (C_max_) of 7 mg·kg^−1^ KM was ~12-fold and ~24-fold higher than that of 0.28 mg·kg^−1^ KM in adult and aged rats, respectively (*P* < 0.01). Time to reach C_max_ (T_max_) for 7 mg·kg^−1^ KM was 4-fold longer in aged rats (*P* < 0.05). The area under the curve (AUC) of 7 mg·kg^−1^ KM was >17-fold and >43-fold higher than those of 0.28 mg·kg^−1^ KM in adult and aged rats, respectively (*P* < 0.01). The half-life (t_1/2_) of 7 mg·kg^−1^ KM was over 4-fold longer than that of 0.28 mg·kg^−1^ KM in adult rats (*P* < 0.01). The t_1/2_ of 1.4 and 7 mg·kg^−1^ KM were 1.5~2-fold longer, than that of 0.28 mg·kg^−1^ KM in aged rats (*P* < 0.05). The clearance rate of 7 mg·kg^−1^ KM was significantly lower in aged than in adult rats (*P* < 0.05). For 7.0 mg·kg^−1^ KM, the C_max_ in aged rats was higher than in adult rats during the T_max_ period (*P* < 0.05). In aged rats, the AUC for KM was >2.5-fold higher (*P* < 0.05) and the t_1/2_ was >60% longer than in adult rats (*P* < 0.05). These results help interpret the pharmacokinetics of KM in aging-associated diseases.

## Introduction

Aging causes a decline in organ function and decreased efficacy of homeostatic systems, ultimately resulting in the alteration of nearly all pharmacokinetic phases (Mangoni and Jackson, 2004). There has been a rapid increase in the rate aging population (Prince et al., 2015). Older individuals frequently suffer from a variety of chronic conditions and are required to consume multiple medications over a long period (Qato et al., 2016). However, the elderly population is often excluded from clinical trials; therefore, evidence and indications for the use of medications in this population are limited (Van Spall et al., 2007).

Koumine (KM), the most abundant alkaloid in *Gelsemium elegans* Benth. (*G. elegans*) has various pharmacological properties and is known to be effective in the treatment of numerous age-associated conditions including postoperative pain (Xiong et al., 2017; Shoaib et al., 2019), inflammation (Yuan et al., 2016; Wu et al., 2020), rheumatoid arthritis (Yang et al., 2016), malignant tumors (Yuan et al., 2019a), nonalcoholic fatty liver disease (Yue et al., 2019), oxidative stress (Yuan et al., 2019b), and neuropathic pain (Jin et al., 2018a; Jin et al., 2018b). However, the pharmacokinetic profile of KM in the elderly or aged animals remains to be fully evaluated.

Pharmacokinetic profiling is vital for understanding the *in vivo* behavior and mechanism of action of a drug. On account of its diverse biological activities, many metabolic and excretion studies in rats (Chen et al., 2013; Wang et al., 2018a; Wang et al., 2018b), pigs (Yang et al., 2018; Liu et al., 2019), and goats (Cao et al., 2020; Zuo et al., 2020) have investigated the role of KM. However, the dose proportionality of KM following oral administration, particularly in aged individuals, remains unknown.

In the current study, we systematically investigated the dose proportionality and pharmacokinetics of orally administered KM in adult (2-month-old) and aged (9-month-old) rats.

## Materials and Methods

### Materials

We isolated KM and the internal standard (IS) gelsemine from *G. elegans*, with a purity of >99.0% (by high-performance liquid chromatography [HPLC]) using our previously established method (Su et al., 2011). We purchased HPLC-grade methanol from Merck (Darmstadt, Germany), formic acid (analytical grade) from Anaqua™ Chemicals Supply (Houston, Texas, USA), and pooled heparinized rat plasma from Nanjing SenBeiJia Biological Technology (Nanjing, Jiangsu, China). Deionized water was generated using a Millipore Direct-Q^®^ water purification system (Millipore, Billerica, MA, USA). All other reagents were of analytical grade.

### Animals

In this study, we obtained 2-month-old adult (average weight: 191.2 ± 4.3 g) and 9-month-old aged (average weight: 598.7 ± 28.2 g) male Sprague-Dawley (SD) rats from Zhejiang Vital River Laboratory Animal Technology (Jiaxing, China; certificate number SCXK2019-0001). The animals were acclimatized with *ad libitum* access to water and food for at least one week prior to experimentation. The environmental conditions were maintained as follows: 20°C–26°C, 40%–70% relative humidity, and 12-h light/dark cycles. The animals were fasted overnight prior to the experiment with *ad libitum* access to water. All experimental procedures and protocols were approved by the Animal Ethics Committee of Fujian Medical University (No. 2017-01) and abided by the Guide for the Care and Use of Laboratory Animals (NIH Publications No. 8023, revised 1978).

### Preparation of Stock Solutions, Calibration Standards, and Quality Control Samples

Stock solutions of KM (1.0 mg·ml^−1^) and IS (1.0 mg·ml^−1^) were prepared separately by dissolving 5 mg of KM and 5 mg of IS in 5 ml methanol-water solution (50:50, *v:v*). They were then stored at −80°C until further use. We prepared standard working solutions by serially diluting the stock solutions with methanol.

We prepared the calibration standard solutions by evaporating working solutions to dryness at 55°C in a centrifugal vacuum concentrator and then mixing with 50 μl of pooled blank SD rat plasma to obtain calibration concentrations ranging from 0.2 to 200 ng·ml^−1^ for KM and 80 ng·ml^−1^ for the IS. Low-, medium-, and high-concentration quality control (QC) samples were prepared at 0.6, 10, and 150 ng·ml^−1^, respectively.

### Chromatography and Tandem Mass Spectrometry

We performed a chromatographic assay using an Agilent 1290 Infinity Liquid Chromatography system (Agilent Technologies, Santa Clara, CA, USA) on a reversed-phase C18 column (Agilent ZORBAX Eclipse XDB-C18: 4.6 × 50 mm, 3.5 μm). This chromatographic system was linked to an AB SCIEX QTRAP^®^ 5500 mass spectrometer (AB SCIEX, Framingham, MA, US) equipped with Turbo Ion Spray™ electrospray ionization (ESI) probes. Data generated from the ultra-performance liquid chromatography-tandem mass spectrometry (UPLC-MS/MS) system was analyzed using the Analyst^®^ 1.6.3 software (AB SCIEX). The mobile phase was composed of methanol and water with 0.1% formic acid at 40°C. The injection volume was 5 μl and flow rate was maintained at 0.4 ml·min^−1^. The gradient program was as follows: (a) 0.0–1.0 min: 20% methanol; (b) 1.0–4.0 min: 20%–95% methanol; (c) 4.0–5.0 min: 95% methanol; (d) 5.0–5.01 min: 95%–20% methanol; and (e) 5.01–10.0 min: 20% methanol. Only the eluent from 2.0–5.0 min was directly passed into the mass spectrometer operated in the positive ESI mode.

N_2_ was used as the nebulizing, curtain, and collision gas in the mass spectrometer. We explored the ion mode with higher detection sensitivity for the parent ion of KM to optimize operating parameters of the MS system. The source parameters including curtain gas, ion source gas 1, ion source gas 2, temperature, and ion spray voltage, were set at 20 psi, 55 psi, 55 psi, 550°C, and 5,500 V, respectively. We performed sequential ramping of the operation potentials to identify optimal parameters, which consisted of declustering potential, entrance potential, collision energy, and collision exit cell potential (181.1 V, 10.0 V, 58.8 V, and 13.0 V for KM and 155.0 V, 10.0 V, 63.6 V, and 13.0 V for IS, respectively). Given that the MS/MS spectra of both KM and IS produced abundant single fragment ions, we used the multiple reaction monitoring mode at *m/z* 307.2→180.1 for KM and *m/z* 323.1→70.1 for the IS with a dwell time of 100 ms.

### Method Validation

We validated the LC-MS/MS method by examining its selectivity, sensitivity, linearity, accuracy, precision, extraction recovery, matrix effect, and stability under various conditions, in accordance with the United States Food and Drug Administration Bioanalytical Method Validation Guidance for Industry (Center for Drug Evaluation and Research, 2018).

#### Selectivity

Selectivity was established by comparing the chromatograms of six individual blank rat plasma samples with the chromatograms of KM- and IS-spiked plasma samples. In addition, the selectivity was verified by chromatographic comparison of plasma samples obtained from the pharmacokinetic study, pre- and post-administration.

#### Linearity and Lower Limit of Quantification

The response was measured as the peak area ratio of KM to IS. We performed a linear regression analysis, where *x* was the KM concentration, *y* was the peak area ratio of KM to IS, and 1/*x* ^2^ was chosen as the weighting factor. The goodness-of-fit of linear regression was described using *r*. We assessed linearity using calibration standards (0.2, 1, 2, 10, 20, 100, and 200 ng·ml^−1^) on three consecutive days. On day 1, five sample replicates were prepared, whereas duplicate samples were used on days 2 and 3. Sensitivity was represented by the lower limit of quantification (LLOQ), which was defined as the lowest level that produced a signal-to-noise ratio not less than 10 with satisfactory accuracy (relative error [RE] of mean analytical recovery between 20 and −20%) and precision (coefficient of variation [CV] ≤ 20%).

#### Precision and Accuracy

We evaluated QC samples at the LLOQ of 0.2 ng·ml^−1^ and QC levels of 0.6, 10, and 150 ng·ml^−1^ for KM in five separate runs within a day to assess intra-day accuracy and precision; we evaluated one series per day on at least three consecutive days to evaluate the inter-day accuracy variation. Accuracy was calculated by presenting the determined concentrations as percentages of the equivalent nominal levels (analytical recovery) and the precision was determined by assessing the CV. The assay was regarded as accurate and precise if the RE of analytical recovery was ±15% and CV ≤15%.

#### Extraction Recovery and Matrix Effects

The extraction recovery (%) was calculated as the peak area ratio between the post-extraction and pre-extraction spiked plasma samples with the same concentration of KM or IS. The matrix effect was assessed with an IS-normalized matrix factor using individual blank plasma samples from six different rats. The matrix factor of KM or IS was defined as the peak area ratio of the spiked post-extraction plasma samples to that of the standard solutions containing the same amount of KM or IS. The IS-normalized matrix factor was calculated by comparing the matrix factor of KM with that of IS. The matrix effect was regarded as insignificant if the CV of the IS-normalized matrix factor was <15%.

#### Stability

The stability of KM in plasma samples was tested at the three QC concentrations while that of IS was tested using three replicates under conditions commonly encountered during sample storage, preparation, and analysis. These included freeze-thaw stability after three cycles (frozen at −20°C and thawed three times), short-term stability (storage for 4 h at 25°C), long-term stability (−80°C for 20 days), and post-preparative stability (storage after sample preparation at 25°C for 48 h). KM or IS was regarded as stable if its peak area deviated by less than 15% of the equivalent freshly prepared samples.

### Administration and Sampling

Eighteen 2-month-old male rats were randomly divided into low-, medium-, and high-dose groups, and administered KM by oral gavage (0.28, 1.4, and 7.0 mg·kg^−1^ for the low-, medium-, and high-dose groups, respectively). Blood samples (300 μl) were collected from the retro-orbital plexus into heparinized microcentrifuge tubes before administration and at 0.033, 0.083, 0.17, 0.33, 0.67, 1, 2, 4, 6, 8, and 12 h post-administration. Rats were fed a standard diet 2 h following dosing. Plasma was immediately obtained from the blood by centrifugation (2,000 *× g* for 5 min). All plasma samples were stored at –80°C until further analysis. The sample size, grouping, drug administration, and sampling of 9-month-old male rats were similar to those of the 2-month-old male rats.

Plasma (50 μl) was then mixed with 20 μl of the IS solution (final concentration: 80 ng·ml^−1^) and evaporated to dryness in a 50-bar centrifugal vacuum concentrator at 1,000 *× g* for 10 min at 55°C. Next, ethyl acetate (400 μl) was added to the samples, which were then vortex-mixed for 3 min and centrifuged at 12,000 *× g* for 10 min at 4°C. The supernatants (380 μl) were carefully transferred into fresh microcentrifuge tubes and evaporated to dryness using 50-bar vacuum centrifugation at 1,000 *× g* for 12 min at 55°C. The residues were reconstituted in 100 μl methanol-water solution (50:50, *v*:*v*), vortex-mixed for 3 min and centrifuged at 12,000 *× g* for 10 min at 4°C. Finally, an aliquot of the supernatant (5 μl) was injected for UPLC-MS/MS analysis.

### Pharmacokinetic Parameters and Statistical Analyses

Primary pharmacokinetic parameters were determined using the non-compartmental model with DAS 3.2 software (Shanghai, China). The maximum plasma concentration (C_max_) and time to reach C_max_ (T_max_) of KM were obtained directly from the actual data. The area under the plasma concentration-time curve (AUC) is defined as the area under the plasma concentration-time curve from zero to the last measured time (AUC_0–t_) or to infinity (AUC_0–∞_) and was determined using the log-linear trapezoidal method. The terminal phase elimination rate (λ_z_) was calculated using the log-linear regression of the plasma concentration data during the terminal phase, and the biological half-life during the elimination phase (t_1/2_) was estimated as ln(2)/λ_z_. The clearance rate (CL) and apparent volume of distribution (V_d_) were determined as the dose/AUC_0–∞_ and CL/λ_z_, respectively (Kim and Baek, 2018). Statistical analysis was performed using two-way analysis of variance with Dunnett’s *post hoc* analysis using IBM SPSS Statistics 23.0 (Armonk, NY, USA). Results shown in tables and figures are presented as the mean ± standard deviation. Differences between groups were considered significant when *P* < 0.05. We plotted the figures using GraphPad Prism 7.0 (San Diego, CA, USA).

## Results

### UPLC-MS/MS Method Development and Optimization

**Figure 1:**
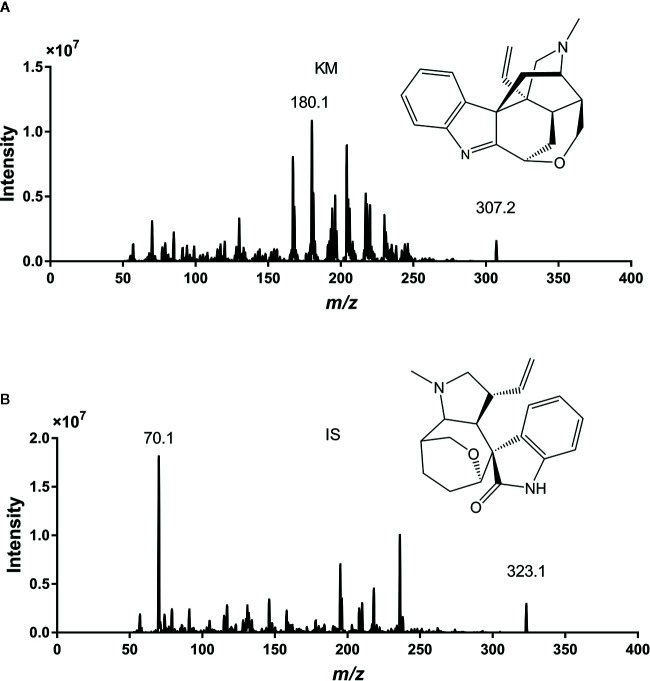
The chemical structures and full-scan precursor ions and products of Koumine (KM) **(A)** and internal standard (IS) **(B)**.

We optimized the UPLC-MS/MS method to quantify KM and the IS in rat plasma. We tuned the full-scan mass spectra and found that the signal intensity of the two analytes was more robust in the positive than in the negative ion mode, most likely because the nitrogen atoms in the alkaloid structure tend to be protonated (Chen et al., 2013; Wang et al., 2018b). Therefore, we adopted the positive ion mode throughout this study. In the full-scan spectrum of precursor ions, the most abundant ions for KM and the IS were protonated [M+H]^+^ at *m/z* 307.2 and 323.1, respectively. Parameters such as desolvation temperature, ESI source temperature, capillary and cone voltage, and flow rate of the desolvation gas and cone gas were optimized to obtain the relative abundance of the most abundant precursor and product ions. During optimization, the multiple reaction monitoring mode was used to improve specificity of detection. The product ion scan spectra of KM and IS are shown in **Figure 1**. The strongest peaks for the quantification of KM and IS corresponded to the precursor-product ion transitions of *m/z* 307.2→180.1 and 323.1→70.1, respectively.

We tested several mobile phase systems to enhance the sensitivity of detection and obtain the ideal peak shape; we explored different combinations of mobile phase systems including 20 mM ammonium acetate solution (with or without 0.1% formic acid and 5% acetonitrile) and methanol, 0.1% formic acid solution and acetonitrile, and 0.1% formic acid solution and methanol to improve peak shape and enhance ESI. Ultimately, we used an Agilent ZORBAX Eclipse XDB-C18 column (4.6 × 50 mm, 3.5 μm) at 40°C with a flow rate set to 0.4 ml·min^−1^ and selected a mobile phase of 0.1% formic acid solution (A) and methanol (B). All analytes were separated rapidly within 4 min without interference from the endogenous components of rat plasma.

### Sample Preparation Development and Optimization

Liquid-liquid extraction (LLE) or a protein precipitation method was explored for the analytes using cold acetonitrile, methanol, and ethyl acetate. We ultimately selected ethyl acetate for LLE owing to its acceptable efficiency with respect to precipitation. The data for the extraction recovery and CV are shown in **Table S1**.

### Method Validation

#### Specificity and Selectivity

**Figure 2:**
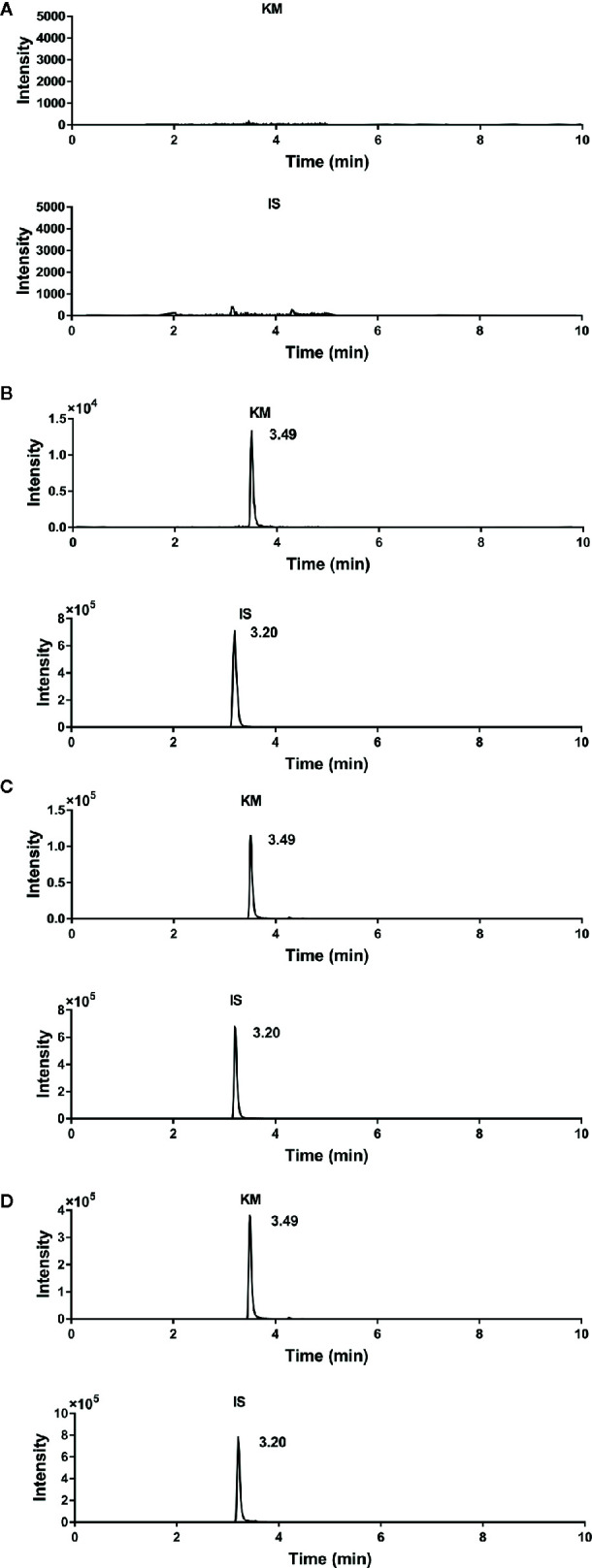
Representative chromatograms of **(A)** blank rat plasma; **(B)** blank rat plasma spiked with Koumine (KM) (final concentration: 2.0 ng·ml^−1^), and internal standard (IS) (final concentration: 80 ng·ml^−1^); **(C)** adult rat plasma collected 0.17 h after a single intragastric administration of 1.4 mg·kg^−1^ KM; and **(D)** aged rat plasma collected 0.17 h after a single intragastric administration of 1.4 mg·kg^−1^ KM.

Under optimal conditions, the retention times of KM and IS were 3.49 and 3.20 min, respectively. The representative chromatograms for blank plasma, plasma spiked with KM and IS, and plasma collected from adult and aged rats 10 min following intragastric administration of 1.4 mg·kg^−1^ KM are shown in **Figure 2**. We observed no interfering peaks at the elution times, which suggested that this method was selective for the determination of KM in rat plasma.

#### Linearity and LLOQ

We analyzed and evaluated the calibration curves for KM. The peak area ratios of KM to IS in rat plasma showed a linear change in the concentration range of 0.2–200 ng·ml^−1^. The optimal linear fit and least-square residuals for the calibration curve were obtained using 1/*x^2^* as the weighting factor. The regression equation and correlation for KM were determined using the equation *y* = 6.15 × 10^−3^ *x* + 1.73 × 10^−3^ (*r* = 0.9990), where *y* represents the peak area ratio of KM to IS and *x* represents the concentration of KM in plasma. Our UPLC-MS/MS method yielded an LLOQ value of 0.2 ng·ml^−1^ for KM with an accuracy (RE) of 1.9%, and intra- and interday precision (CV) of <18.0% when measured on three consecutive days (*n* = 5).

#### Precision and Accuracy

The intra- and inter-day precisions ranged from 3.6% to 10.7% and 8.0% to 11.7%, respectively (**Table 1**). The accuracy (RE) ranged from −8.8% to 4.0%. These results demonstrated the suitability of the assay in terms of accuracy and precision.

**Table 1:** Precision and accuracy of the determination of Koumine (KM) concentration in rat plasma (*n* = 5).

Spiked Concentration (ng·ml^−1^)	Accuracy	Intra-day precision	Inter-day precision
	RE (%)	CV (%)	CV (%)
0.2	1.9	10.3	17.1
0.6	−3.4	10.7	11.7
10	4.0	3.9	8.3
150	−8.8	3.6	8.0

#### Extraction Recovery and Matrix Effect

The mean extraction recoveries of KM were 59.6%, 56.9%, 70.1%, and 73.0% with an LLOQ of 0.2 ng·ml^−1^ and QC levels of 0.6, 10, and 150 ng·ml^−1^ for KM (all CVs <9%; **Table 2**). The recovery of IS (80 ng·ml^−1^) was 62.4% (CV 7.6%). The matrix effects for the QC samples were 98.0%–108.0% for KM (CV <5%). We observed no noticeable matrix effects and considered the protocol to be reliable for plasma bioanalysis.

**Table 2:** Matrix effects and extraction recovery of Koumine (KM) in rat plasma (*n* = 6).

Spiked Concentration(ng·ml^−1^)	Matrix effect		Extraction recovery	
	Mean (%)	CV (%)	Mean (%)	CV (%)
0.2	98.0	4.4	59.6	1.9
0.6	100.0	2.4	56.9	8.4
10	100.0	1.6	70.1	4.4
150	108.0	2.2	73.0	5.5
IS	–	–	62.4	7.6

#### Stability

The stability data for repeated freezing-thawing, long-term storage, short-term storage, and post-preparation of KM and IS are shown in **Table 3**. KM and IS were sufficiently stable for bioanalysis under different conditions (RE < ±15%).

**Table 3:** Stability of Koumine (KM) in rat plasma under different conditions (*n* = 3).

Spiked Concentration (ng·ml^−1^)	3 Freeze-thaw cycles	Short-term storage	Long-term storage	Post-preparative
	RE (%)	RE (%)	RE (%)	RE (%)
0.2	−4.2	1.3	3.3	−1.6
0.6	3.3	−1.7	11.0	0.4
10	−11.7	−13.7	−5.8	9.6
150	−9.6	−9.3	−6.0	−4.5
IS	−3.1	1.9	−11.7	5.4

### Dose Proportionality Pharmacokinetics of KM in Adult Rats

**Figure 3:**
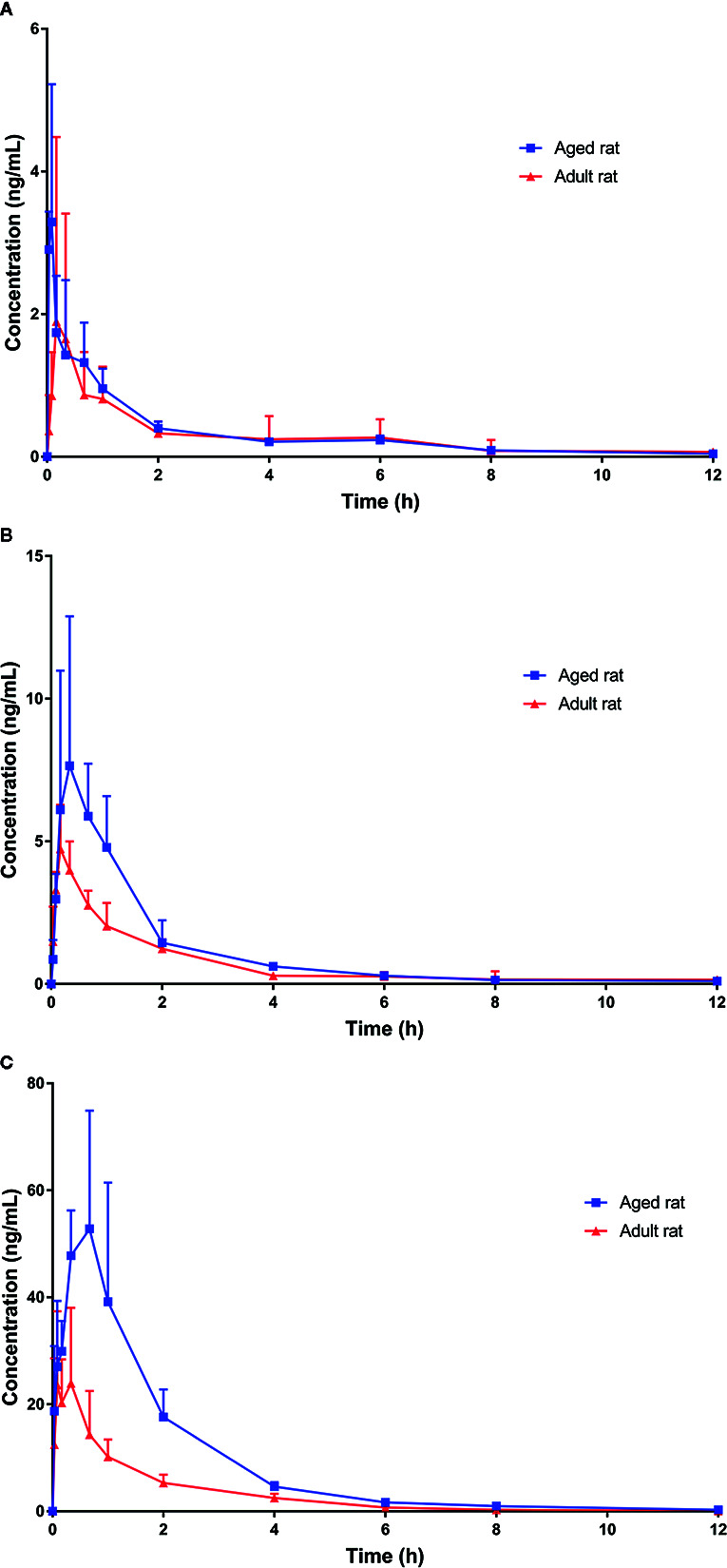
Plasma concentration-time profiles of Koumine (KM) following intragastric administration of **(A)** 0.28 mg·kg^−1^; **(B)** 1.4 mg·kg^−1^; and **(C)** 7.0 mg·kg^−1^ KM. Data are presented as the mean ± standard deviation (*n* = 6).

We successfully used our UPLC-MS/MS method to investigate the pharmacokinetics of orally administered KM (single doses of 0.28, 1.4, or 7.0 mg·kg^−1^) using rat plasma. The basis for using this dose in rats was the anti-allodynic and neuroprotective effects of KM established in a previous study (Ling et al., 2014). Based on these data, three doses were selected for pharmacokinetic studies on aging-associated diseases during preclinical studies of KM. Since our preliminary experiments and published studies indicated that KM was rapidly absorbed, we added an early time-point (0.033 h) to the absorption phase (Wang et al., 2018b). Furthermore, we added two additional time-points near the C_max_ in the equilibrium phase to more accurately assess plasma concentration and other parameters (Wang et al., 2018b). The pharmacokinetic parameters calculated using the non-compartmental model are listed in **Table 4**. The mean plasma concentration-time curves of KM are shown in **Figure 3**. We detected KM in rat plasma within 0.033 h; approximately 0.5 h later, the plasma concentration rapidly declined followed by a slower decrease after ~2 h. The C_max_ of the 7 mg·kg^−1^ dose was approximately 12-fold higher than that of the 0.28 mg·kg^−1^ dose (29.98 ± 13.39 versus 2.60 ± 1.67 ng·ml^−1^; *P* < 0.01; **Table 4**). In addition, the AUC_0–t_ and AUC_0–∞_ of the 7 mg·kg^−1^ dose were >17-fold higher than that of the 0.28 mg·kg^−1^ dose (AUC_0–t_: 49.33 ± 25.85 versus 2.81 ± 1.14 ng·L·h^−1^; AUC_0–∞_: 49.78 ± 25.78 versus 2.89 ± 1.10 ng·L·h^−1^; all *P* < 0.01; **Table 4**). The t_1/2_ of the orally administered 7 mg·kg^−1^ KM dose was 62% longer than that of the 0.28 mg·kg^−1^ KM dose in adult rats (1.26 ± 0.17 versus 0.78 ± 0.17 h; *P* < 0.01; **Table 4**). The V_d_ of KM was significantly higher for the 7 mg·kg^−1^ dose than for the lower doses (*P* < 0.05; **Table 4**). T_max_ and CL values did not significantly differ among the three doses (*P* > 0.05).

**Table 4:** Pharmacokinetic parameters of orally administered Koumine (KM) (0.28, 1.4, or 7.0 mg·kg^−1^) in 2-month-old adult rats or 9-month-old aged rats (*n* = 6).

Parameters^a^	Administered dose					
	0.28 mg·kg^−1^		1.4 mg·kg^−1^		7.0 mg·kg^−1^	
	Adult rat	Aged rat	Adult rat	Aged rat	Adult rat^b^	Aged rat
C_max_ (ng·ml^−1^)	2.60 ± 1.67	3.00 ± 1.82	5.49 ± 0.94	7.88 ± 5.29	29.98 ± 13.39^##^	72.60 ± 20.63^*,††^
T_max_ (h)	0.46 ± 0.37	0.09 ± 0.06	0.23 ± 0.12	0.33 ± 0.00	0.23 ± 0.12	0.39 ± 0.25^†^
AUC_(0-t)_ (ng·h·ml^−1^)	2.81 ± 1.14	2.82 ± 0.97	7.97 ± 1.45	11.88 ± 5.22	49.33 ± 25.85^##^	130.14 ± 38.09^*,††^
AUC_(0-∞)_ (ng·h·ml^−1^)	2.89 ± 1.10	2.98 ± 0.92	8.22 ± 1.65	12.65 ± 5.14	49.78 ± 25.78^##^	130.90 ± 37.95^*,††^
λ_z_ (h^-1^)	0.91 ± 0.18	0.73 ± 0.27	0.66 ± 0.11^#^	0.41 ± 0.09^*,†^	0.55 ± 0.07^##^	0.34 ± 0.05^**,†^
t_1/2_ (h)	0.78 ± 0.17	1.06 ± 0.40	1.07 ± 0.18	1.75 ± 0.39^*,†^	1.26 ± 0.17^##^	2.08 ± 0.29^**,††^
V_d_ (L·kg^−1^)	115.33 ± 25.83	161.86 ± 96.87	266.03 ± 39.88	327.63 ± 173.29	313.19 ± 157.76^#^	180.18 ± 90.86
CL (L·h^−1^·kg^−1^)	106.64 ± 34.74	100.12 ± 26.72	174.83 ± 29.50	122.99 ± 41.59	166.17 ± 68.33	58.63 ± 23.76^*^

^a^C_max_, maximum plasma concentration; T_max_, time to reach C_max_; AUC, area under the plasma, concentration-time curve; λ_z_, terminal phase elimination rate; t_1/2_, biological half-life in the elimination phase; V_d_, apparent volume of distribution; CL, clearance rate.

^b*^, P < 0.05 and ^**^, P < 0.01 compared to adult rats; ^#^, P < 0.05 and ^##^, P < 0.01 compared to recipients of 0.28 mg·kg^−1^ KM among adult rat groups; ^†^, P < 0.05 and ^††^, P < 0.01 compared to recipients of 0.28 mg·kg^−1^ KM among aged rat groups.

### Dose Proportionality Pharmacokinetics of KM in Aged Rats

Pharmacokinetic parameters of the 9-month-old rats are listed in **Table 4**. The mean plasma concentration-time curves for KM are illustrated in **Figure 3**. The C_max_ of the 7 mg·kg^−1^ dose was approximately 24-fold higher than that of the 0.28 mg·kg^−1^ dose (72.60 ± 20.63 versus 3.00 ± 1.82 ng·ml^−1^; *P* < 0.01; **Table 4**). The T_max_ of the orally administered 7 mg·kg^−1^ KM dose was over 4-fold longer than that of the 0.28 mg·kg^−1^ dose in aged rats (0.39 ± 0.25 versus 0.09 ± 0.06 h; *P* < 0.05; **Table 4**). Furthermore, the AUC_0–t_ and AUC_0–∞_ of 7 mg·kg^−1^ KM were >43-fold higher than those for the 0.28 mg·kg^−1^ dose (AUC_0–t_: 130.14 ± 38.09 versus 2.82 ± 0.97 ng·L·h^−1^; AUC_0–∞_: 130.90 ± 37.95 versus 2.98 ± 0.92 ng·L·h^−1^; all *P* < 0.01; **Table 4**). The t_1/2_ of 1.4 and 7 mg·kg^−1^ KM were >1.5-fold and nearly 2-fold longer, respectively, than that of orally administered 0.28 mg·kg^−1^ KM in aged rats (1.75 ± 0.39 versus 2.08 ± 0.29 versus 1.06 ± 0.40 h, respectively; all *P* < 0.05; **Table 4**). The CL of KM was significantly lower for the 7 mg·kg^−1^ dose than for the lower doses (*P* < 0.05; **Table 4**). The V_d_ values did not significantly differ among the three doses (*P* > 0.05).

### Comparison of Pharmacokinetics of KM Between Adult and Aged Rats

For the 7.0 mg·kg^−1^ dose, the C_max_ of KM in aged rats was significantly higher than in adult rats during the T_max_ period (72.60 ± 20.63 versus 29.98 ± 13.39 ng·ml^−1^; *P* < 0.05; **Table 4**). Thus, in aged rats, compared to adult rats, the AUC_0–t_ and AUC_0–∞_ for KM were >2.5-fold higher (AUC_0–t_: 130.14 ± 38.09 versus 49.33 ± 25.85 ng·L·h^−1^; AUC_0–∞_: 130.90 ± 37.95 versus 49.78 ± 25.78 ng·L·h^−1^; all *P* < 0.05; **Table 4**) and the t_1/2_ was significantly prolonged by over 60% (all *P* < 0.05; **Table 4**). The systemic CL of KM was significantly lower in aged rats than in adult rats (58.63 ± 23.76 versus 166.17 ± 68.33 L·h^−1^·kg^−1^; *P* < 0.05; **Table 4**). The T_max_ and V_d_ values did not significantly differ among the three doses in adult and aged rats (*P* > 0.05).

## Discussion

To the best of our knowledge, this is the first report assessing the dose-dependent pharmacokinetics of KM following oral administration. We developed a sensitive UPLC-MS/MS method requiring a small volume of plasma to determine KM levels in a pharmacokinetic study involving adult and aged rats. The basis for using this dose was the anti-allodynic and neuroprotective effects of KM established in a previous study (Ling et al., 2014). At doses of 0.28, 1.4, and 7 mg·kg^−1^, KM alleviated the damage to myelin structures and vacuolar defects, significantly mitigated lamellar separation, increased the size of myelin and axons, and normalized Schwann cells (Ling et al., 2014). Considering the difficulty associated with blood collection in aged rats, this new method is beneficial as it requires only 50 μl of plasma unlike previously reported methods that require 100 μl (Chen et al., 2013; Wang et al., 2018a; Wang et al., 2018b). Moreover, the LLOQ of the new method was 0.2 ng/ml, which implies more sensitivity than the LLOQ achieved in some previous studies (Chen et al., 2013; Xu et al., 2013) and similar to that reported in others (Wang et al., 2018a; Wang et al., 2018b; Yang et al., 2018). This facilitates the detection of KM in the low-dose group 12 h following administration. The linear range is wider than that of previously reported methods (Chen et al., 2013; Wang et al., 2018a; Wang et al., 2018b). Specifically, the linear range for our method was 1,000-fold. Further, the upper limit of quantification was increased to 200 ng/ml, which ensured that the pharmacokinetic detection needs for aged rats with large individual differences were met.

Few studies have assessed the dose-dependent pharmacokinetics of KM following oral administration. We had previously performed a pharmacokinetic analysis of KM in rat plasma following an intragastric administration of 15 mg·kg^−1^ KM powder (purity >99.1%) (Xu et al., 2013), which revealed a T_max_ and t_1/2_ of 19.95 ± 0.53 min (equivalent time in hours: 0.33 ± 0.01 h) and 234.11 ± 17.34 min (equivalent time in hours: 3.90 ± 0.29 h), respectively. Wang et al. investigated the pharmacokinetics of KM in rat plasma following oral administration of a *G. elegans* extract (single dose: 10 mg·kg^−1^) equivalent to 1.22 mg·kg^−1^ KM (Wang et al., 2018a; Wang et al., 2018b). They observed a T_max_ and t_1/2_ of 0.28 ± 0.07 h and 1.60 ± 0.21 h, respectively. Their results were consistent with our findings in adult rats. According to the experimental parameters reported by a different research group, the t_1/2_ of KM following intragastric administration is greater than that following injection, presumably as it takes longer for the drug to be absorbed *via* the duodenum, jejunum, and ileum after oral administration (Chen et al., 2013; Shi et al., 2017; Wang et al., 2018a). Although KM bioavailability has yet to be accurately reported, it can be roughly calculated from the combined experimental parameters reported by Wang et al. as only 1% (Wang et al., 2018a; Wang et al., 2018b).

Limited data are available on the pharmacokinetics of KM in aged rats; however, rodents older than 9 months commonly show signs of the later stages of aging (Liu et al., 2016). For the 1.4 and 7.0 mg·kg^−1^ orally administered doses, we found that the T_max_ of KM occurred approximately 0.3 h later in aged rats. This difference likely resulted from age-associated changes in the gastrointestinal tract and hepatorenal function, including decreases in gastric emptying time, gastrointestinal peristalsis, bowel surface area, and splanchnic blood flow (Wang et al., 2018a; Zeng et al., 2019). When the KM dose was increased from 0.28 to 1.4 mg·kg^−1^ and from 1.4 to 7.0 mg·kg^−1^, the AUC_0–t_ and AUC_0–∞_ did not increase proportionally with the dose (**Table 4**), which was indicative of the nonlinearity of the pharmacokinetics for the 7.0 mg·kg^−1^ dose. Further experiments are warranted to confirm and explain this phenomenon. We found a considerably higher C_max_ of KM, prolonged t_1/2_, lower systemic CL, and unchanged T_max_ in aged rats. We speculated that this might be due to binding by plasma proteins or the saturated metabolism of KM (Zhang et al., 2015). One reason for this might be the reduction in plasma albumin concentrations of ~10–15% in the elderly (Greenblatt, 1979; Reeve et al., 2015). At 0.28 and 1.4 mg·kg^−1^ doses of KM, the plasma protein concentrations might be sufficient to bind KM to form a bound drug. Therefore, no significant differences were found in the plasma concentration of free fractions of KM and AUC between the aged and adult rats. However, when the dose of KM increased to 7.0 mg·kg^−1^, rats with relatively lower plasma protein concentrations theoretically had an increased free fraction of KM, partly leading to a significantly higher AUC in the aged rats compared to that in adult rats. The other possible reason might be hepatic metabolism. Based on *in vitro* and *in vivo* results, the observed reduction in the metabolism of drugs, which undergo phase I metabolism, is most likely due to the reduced blood flow and liver mass, rather than a reduction in the expression or activity of the CYP enzymes (Le Couteur et al., 2005; Hilmer et al., 2007; Reeve et al., 2015). Both hepatic blood flow and liver mass were reduced with age (Wilkinson, 1997). Hepatic blood flow was reduced in the aged population by ~20%–50%, with a similar reduction in liver mass (Cotreau et al., 2005; Reeve et al., 2015). This reduction in hepatic blood flow affects the rate of drug metabolism differently, depending on their extraction ratio (the ratio of hepatic clearance in relation to hepatic blood flow) (Tan et al., 2015). Unfortunately, the extraction ratio of KM remains unknown, making it impossible to assess the effect of reduced liver blood flow on KM. We also observed that a reduction in the clearance of KM might be due to reduced liver mass (and therefore a decrease in the surface area and enzymes for metabolism). Hindrance in the process of oxygen transfer into hepatocytes significantly impacts all enzymatic processes that are oxygen dependent, including CYP pathways, which are particularly dependent on oxygen as a co-substrate (Reeve et al., 2015). Age-related pseudo-capillarization might result in reduced oxygen availability within the hepatocytes and thus limit CYP reactions. This has been proposed as a reason for a reduction in the metabolism of drugs (McLean and Le Couteur, 2004). Similarly, at low and medium doses of KM, the capacity of hepatic metabolism might be sufficient to metabolize KM. Therefore, no significant difference was found in the plasma concentration of KM and the AUC between the aged and adult rats. However, when the KM dose increased to 7.0 mg·kg^−1^, phase I metabolism was saturated, partly leading to significantly higher AUC values in the aged rats compared to those in the adult rats. When applying a high dose of KM to the aged population, special attention should be paid to the plasma drug concentration and its related toxicity.

The CL of KM was lower in aged rats than in adult rats and was accompanied by a higher t_1/2_. In addition, the C_max_ of KM was increased nearly 2.5-fold, with a >2.5-fold higher AUC_0–t_ and AUC_0–∞_. There are several possible explanations for the differences in the pharmacokinetics of KM in aged rats. First, the increased plasma concentrations of KM due to the low CL in the aged rats could be either or partly due to the altered activity of CYP450, resulting in slower formation of metabolites and faster metabolism saturation or binding by plasma proteins (Zhang et al., 2015; Hu et al., 2017; Wang et al., 2019). CYP3A4/3A5 may be the main enzyme responsible for KM metabolism by the liver microsomes; the KM metabolic pathway in liver microsomes involves oxidization, demethylation, and dehydrogenation (Zhang et al., 2013; Hu et al., 2017; Xiao et al., 2017; Zuo et al., 2020). In addition, aged rats may have lower hepatic, NADPH-dependent, and microsomal metabolic enzyme activity (Schmucker, 1985). It is also possible that slower gastrointestinal peristalsis and gastric emptying may prolong gut exposure to KM and lead to more complete absorption (Xu et al., 2013). Lastly, the altered KM kinetics may be due to modulation of the activity of multidrug transporter P-glycoproteins including the hepatic uptake transporters, organic anion transporting polypeptide 1B1 (OATP1B1) or OATP1B3; inhibition of the activity of these plasma membrane efflux pumps may alter the AUC of KM (Niemi et al., 2011; Hu et al., 2017).

There is limited direct evidence that CYP3A activity changes with age. However, there is considerable data to suggest that drug metabolism in the liver decreases with age, not because of changes in enzyme activity, but possibly due to a decrease in liver mass (Schmucker, 1985). Furthermore, based on our previous study, KM is eliminated most slowly from human liver microsomes and reduces by nearly 50% of the degradation rate in rats (Wei et al., 2016; Wei et al., 2017). Therefore, although this study may not necessarily predict the pharmacokinetic changes of KM in the elderly human population, it suggests slower KM pharmacokinetics in the elderly population.

We established, validated, and applied a sensitive UPLC-MS/MS method requiring only a small plasma sample for the quantification of orally administered KM in adult and aged rats. We systematically investigated and calculated the pharmacokinetic parameters of KM after administration of three doses and observed age-related changes in the behavior of KM *in vivo*. Aging can lead to pharmacodynamic changes in KM, which can result in increased adverse drug reactions especially with high doses of KM. These results provide a reference for the safe and effective use of KM and in further pharmacological studies on KM in aging-associated diseases or clinical studies.

## Data Availability Statement

The raw data supporting the conclusions of this article will be made available by the authors, without undue reservation.

## Ethics Statement

The animal study was reviewed and approved by the Animal Ethics Committee of Fujian Medical University (No. 2017-01).

## Author Contributions

LXY, YX, and CXY conceived and designed the experiments. LXY, DXC, LFC, SHZ, and HHH performed the experiments. LXY and YPS analyzed the data. LXY and YX wrote the paper. All authors contributed to the article and approved the submitted version.

## Funding

We gratefully acknowledge the support of the Natural Science Foundation of Fujian (grant numbers, 2018J01815 and 2019J01307), the Science Foundation for Young Scholars and Teacher of Fujian (grant number, JAT160203), the Central Financial Support Universities Funds of China (grant number, 2018L3008), the Drug Innovation Major Project of China (grant number, 2018ZX09711001-003-024), and the Industry-University-Research Cooperation Project of Fujian Province (grant number, 2017Y4007).

## Conflict of Interest

The authors declare that the research was conducted in the absence of any commercial or financial relationships that could be construed as a potential conflict of interest.
