# Supplementary material for: Orally Administered Koumine Persists Longer in the Plasma of Aged Rats Than That of Adult Rats as Assessed by Ultra-Performance Liquid Chromatography-Tandem Mass Spectrometry
Source: Front Pharmacol. 2020 Jul 21;11:1113. doi: 10.3389/fphar.2020.01113 (PMC7385321; doi:10.3389/fphar.2020.01113)
Supplement: Supplementary file 1 [file DataSheet_1.pdf]

Supplementary Materials

S1: Sample preparation development and optimization

The plasma (50 μL) was then mixed with 20 μL IS solution (final concentration: 80 ng·mL^{-1}) and evaporated to dryness in a 50-bar centrifugal vacuum concentrator at 1,000 × *g* for 10 min at 55 °C. Next, solvent (400 μL) was added to the samples, which were then vortex-shaken for 3 min, and centrifuged at 12,000 × *g* for 10 min at 4 °C. We then carefully transferred the supernatants (380 μL) into fresh microcentrifuge tubes and evaporated them to dryness by 50-bar vacuum centrifugation at 1,000 × *g* for 12 min at 55 °C. The residues were then reconstituted in 100 μL methanol-water solution (50:50, *v*/*v*), vortex-mixed for 3 min and centrifuged at 12,000 × *g* for 10 min at 4 °C. Finally, an aliquot of supernatant (5 μL) was injected for ultra-performance LC (UPLC)-MS/MS analysis. The data for the extraction recovery and CV are shown in Table S1.

Table S1. The extraction recovery and CV for both LLE and PPM

Spiked Concentration	Cold acetonitrile		Methanol		Ethyl acetate	
(ng·mL^{-1})	Mean (%)	CV (%)	Mean (%)	CV (%)	Mean (%)	CV (%)
0.6	51.2	3.3	59.9	18.9	56.9	8.4
10	38.1	2.4	40.8	3.09	70.1	4.4
150	46.0	3.3	48.3	6.2	73.0	5.5
